# End-tidal carbon dioxide levels predict cardiac arrest

**DOI:** 10.1186/cc9500

**Published:** 2011-03-11

**Authors:** H Manyam, P Thiagarajah, G Patel, R French, M Balaan

**Affiliations:** 1 Allegheny General Hospital, Pittsburgh, PA, USA

## Introduction

End-tidal carbon dioxide (CO_2_) correlates with cardiac output during cardiopulmonary resuscitation (CPR) in cardiac arrest patients. Increasing CO_2 _during CPR can also indicate the return of spontaneous circulation.

## Methods

CO_2 _was continuously monitored and recorded every 4 hours in 43 patients who were intubated and on vasopressor medications.

## Results

Mean CO_2 _values were significantly higher in normal patients when compared with those in patients who had a cardiac arrest (30.18 ± 4.93 vs. 17.45 ± 4.76; *P *< 0.001). CO_2 _levels were significantly lower in cardiac arrest patients when compared with hypotensive patients 1, 2, 3, and 4 hours prior to a cardiac arrest (see Table 1). CO_2 _levels were significantly lower in cardiac arrest patients when compared with patients who were acutely withdrawn from care 1, 2, 3, and 4 hours prior to the event (see Table 2).

**Table 1:** End-tidal CO_2 _5 hours prior to cardiac arrest compared with hypotension

Hour prior	Arrest	Hypotension	*P *value
1	16.50	20.67	0.013
2	16.25	21.83	0.013
3	16.50	22.67	0.002
4	16.75	21.33	0.024
5	21.25	21.33	0.99

**Table 2:** End-tidal CO_2 _5 hours prior to cardiac arrest versus acute withdrawal of care

Hour prior	Arrest	Acute withdrawal of care	*P *value
1	16.50	23.29	0.016
2	16.25	24.43	0.001
3	16.50	25.29	< 0.001
4	16.75	26	< 0.001
5	21.25	24.86	0.43

## Conclusions

CO_2 _levels decrease prior to cardiac arrest and are signi-ficantly lower than prior to hypotensive or acute withdrawal of care events. Further study needs to be done on a larger scale to see whether these results hold true.

